# Low sugar and the drive to breathe: Is insulin another adequate stimulus for the carotid body?

**DOI:** 10.1113/EP091008

**Published:** 2023-01-18

**Authors:** Andrew P. Holmes, Prem Kumar

**Affiliations:** ^1^ School of Biomedical Sciences Institute of Clinical Sciences University of Birmingham Edgbaston Birmingham UK

**Keywords:** adrenaline, carotid body, hyperpnoea, hypoglycaemia, insulin

1

Since its correct identification as a sensory organ by De Castro in the 1920s, carotid body research has focused on two key areas, namely its transduction process(es) and its (patho)physiological function. A century later, definitive answers to both remain elusive. One reason might be uncertainty around its adequate stimulus. It is well established that the carotid body is the primary chemoreceptor for systemic hypoxia which, upon stimulation, activates critical cardiovascular and respiratory reflexes to preserve sufficient O_2_ delivery to the brain and vital organs. However, in addition to hypoxia, it is clear that the carotid body can sense a multitude of other potentially harmful stimuli, including hypercapnia, acidosis, systemic inflammation, a rise in temperature and an increase in osmolarity. Thus, perhaps the carotid body is better viewed as a polymodal receptor subserving a generalized function during an acute threat to survival?

Another stressful and potentially life‐threatening stimulus is hypoglycaemia, most commonly occurring in (but not limited to) diabetic patients, owing to disproportionate insulin administration. In these situations, carotid body activation could play an important role in restoring plasma glucose through a reflex increase in adrenaline secretion from the adrenal medulla, promoting glycogenolysis and glucose release from the liver. In addition, metabolism is elevated, and thus another (perhaps more important?) function of carotid body stimulation during hypoglycaemia is to induce a hyperpnoea to counteract the rise in CO_2_ production, thereby preserving normal arterial pH. Indeed, the absence of carotid body activity‐induced hyperpnoea during hypoglycaemia greatly increases the risk of severe hypercapnia and systemic acidosis (Bin‐Jaliah et al., 2004). However, despite a clear functional importance, a major controversy remains regarding the adequate stimulus for the carotid body during hypoglycaemia.

In this issue of *Experimental Physiology*, Baby and co‐workers provide direct evidence, in vivo, that insulin administration increases carotid sinus nerve activity in dogs (Baby et al., 2023). Using whole‐body physiological measurements, the authors demonstrate concomitant changes in heart rate, arterial blood pressure and minute ventilation, although the increased ventilation was not sufficient to prevent a substantial elevation in the arterial partial pressure of CO_2_ and fall in arterial pH. The authors suggest that their findings support the idea that insulin itself can stimulate the carotid body and lead to important reflex changes in cardiovascular and respiratory function.

In their initial experiments, the authors report that intracarotid administration of a single bolus dose of insulin leads to a sustained rise in carotid body chemoafferent activity (Baby et al., 2023). These elegant in vivo electrophysiological measurements show that the rise in discharge frequency is somewhat delayed, being observed ∼2–5 min after insulin administration. The discharge then increases further, peaking at ∼20 min post‐administration. This elevation is accompanied by a rise in minute ventilation and a robust elevation in arterial blood pressure. These data are consistent with previous *ex vivo* experiments that identified the presence of insulin receptors on carotid body type I cells and insulin‐mediated rises in intracellular Ca^2+^ and neurotransmitter release (Ribeiro et al., 2013). What is still to be revealed is the exact mechanism linking insulin receptor stimulation to type I cell depolarization and opening of L‐type Ca^2+^ channels. The delay in the in vivo carotid sinus nerve response to insulin might be indicative of a relatively slow signalling cascade or that it takes time for insulin to accumulate to sufficient levels within the carotid body to activate type I cells. Alternatively, it could be that other stimuli, emerging as a consequence of insulin administration, are required to achieve full carotid body activation. As expected, insulin administration also led to a moderate/severe hypoglycaemia, which could act as the adequate stimulus (Pardal & Lopez‐Barneo, 2002). In addition, it is well known that concentrations of the counter‐regulatory hormone adrenaline increase in blood during hypoglycaemia, and previous evidence has shown that the carotid body can be stimulated by adrenaline (Thompson et al., 2016). The carotid body type I cell is known to express β_1_‐ and β_2_‐adrenoceptors, and both hypoglycaemia and adrenaline stimulation of ventilation are carotid body and β‐adrenoceptor dependent (Thompson et al., 2016). Therefore, the total increase in carotid sinus nerve activity observed here by Baby et al. (2023), which peaked ≥20 min after the initial insulin administration, could be partly or highly dependent on the emergence of hypoglycaemia, the steadily rising levels of adrenaline and the increase in metabolic rate. Using the model described by Baby et al. (2023), it would be interesting to determine whether insulin still augments chemoafferent activity to the same extent during a euglycaemic clamp and thereby quantify the relative contribution of insulin and adrenaline (and possibly other stimuli) in eliciting carotid body activation and hyperpnoea during hypoglycaemia.

Interestingly, this new study also shows that stimulation of carotid body chemoafferent activity by sodium cyanide (a well‐known mitochondrial inhibitor and carotid body chemostimulant) is enhanced by insulin‐induced hypoglycaemia (Baby et al., 2023). Likewise, the rise in ventilation seen with sodium cyanide is exaggerated after insulin administration and emergence of hypoglycaemia. This raises the intriguing possibility that there is significant stimulus interaction between insulin/hypoglycaemia and mitochondrial inhibition within the carotid body. Given that mitochondrial inhibition is commonly considered to underpin hypoxic signalling in the carotid body, we could go a step further and suggest that insulin/hypoglycaemia might well act to modify the carotid body O_2_ sensitivity. Thus, the present work should promote many other studies aiming to characterize the importance of insulin and hypoglycaemia in determining carotid body function and cardiorespiratory control in physiology and pathology. It is tempting to suggest that targeting insulin signalling in the carotid body could be a new clinical approach to treat cardiovascular–respiratory pathology associated with carotid body hyperactivity. Before this, however, more work is needed to establish the precise cellular mechanisms associated with insulin signalling in the development of carotid body hyperactivity, particularly in diseases associated with chronic hyperinsulinaemia. This will certainly be an interesting avenue for future exploration and is of major translational importance.

## AUTHOR CONTRIBUTIONS

Andrew P. Holmes and Prem Kumar conceived the idea and wrote the first draft of the manuscript. Andrew P. Holmes and Prem Kumar approved the final version submitted for publication and agree to be accountable for all aspects of the work in ensuring that questions related to the accuracy or integrity of any part of the work are appropriately investigated and resolved. Both persons designated as authors qualify for authorship, and all those who qualify for authorship are listed.

## CONFLICT OF INTEREST

None declared.
